# Optimizing Existing Mental Health Screening Methods in a Dementia Screening and Risk Factor App: Observational Machine Learning Study

**DOI:** 10.2196/31209

**Published:** 2022-03-22

**Authors:** Narayan Kuleindiren, Raphael Paul Rifkin-Zybutz, Monika Johal, Hamzah Selim, Itai Palmon, Aaron Lin, Yizhou Yu, Ali Alim-Marvasti, Mohammad Mahmud

**Affiliations:** 1 Mindset Technologies Ltd London United Kingdom; 2 Imperial College School of Medicine, Faculty of Medicine Imperial College London London United Kingdom; 3 Medical School University College London London United Kingdom; 4 Medical School University of Michigan Ann Arbor, MI United States; 5 Medical School University of Birmingham Birmingham United Kingdom; 6 Queen Square Institute of Neurology University College London London United Kingdom; 7 Department of Brain Sciences Imperial College London London United Kingdom

**Keywords:** depression, anxiety, screening, research method, questionnaire, precision, dementia, cognition, risk factors, machine learning, prediction

## Abstract

**Background:**

Mindstep is an app that aims to improve dementia screening by assessing cognition and risk factors. It considers important clinical risk factors, including prodromal symptoms, mental health disorders, and differential diagnoses of dementia. The 9-item Patient Health Questionnaire for depression (PHQ-9) and the 7-item Generalized Anxiety Disorder Scale (GAD-7) are widely validated and commonly used scales used in screening for depression and anxiety disorders, respectively. Shortened versions of both (PHQ-2/GAD-2) have been produced.

**Objective:**

We sought to develop a method that maintained the brevity of these shorter questionnaires while maintaining the better precision of the original questionnaires.

**Methods:**

Single questions were designed to encompass symptoms covered in the original questionnaires. Answers to these questions were combined with PHQ-2/GAD-2, and anonymized risk factors were collected by Mindset4Dementia from 2235 users. Machine learning models were trained to use these single questions in combination with data already collected by the app: age, response to a joke, and reporting of functional impairment to predict binary and continuous outcomes as measured using PHQ-9/GAD-7. Our model was developed with a training data set by using 10-fold cross-validation and a holdout testing data set and compared to results from using the shorter questionnaires (PHQ-2/GAD-2) alone to benchmark performance.

**Results:**

We were able to achieve superior performance in predicting PHQ-9/GAD-7 screening cutoffs compared to PHQ-2 (difference in area under the curve 0.04, 95% CI 0.00-0.08, *P*=.02) but not GAD-2 (difference in area under the curve 0.00, 95% CI –0.02 to 0.03, *P*=.42). Regression models were able to accurately predict total questionnaire scores in PHQ-9 (R^2^=0.655, mean absolute error=2.267) and GAD-7 (R^2^=0.837, mean absolute error=1.780).

**Conclusions:**

We app-adapted PHQ-4 by adding brief summary questions about factors normally covered in the longer questionnaires. We additionally trained machine learning models that used the wide range of additional information already collected in Mindstep to make a short app-based screening tool for affective disorders, which appears to have superior or equivalent performance to well-established methods.

## Introduction

Depression was among the 12 modifiable dementia risk factors identified by the Lancet commission [1]. The relationship between depression and dementia is complex, with depression being a risk factor for dementia, a prodromal symptom [2] and a differential diagnosis known as pseudodementia [3]. Anxiety is a highly comorbid condition with depression and is an important feature in the diagnosis of dementia [4]. Anxiety has independent effects on cognition [5] and plays a role in driving health-seeking behavior in individuals without deficits [6]. Furthermore, depression and anxiety symptoms are common in older adults with an estimated prevalence of around 13% [7]. Therefore, appropriate screening for depression and anxiety is of importance when screening for dementia. Mindstep is a new app that aims to holistically screen for cognitive impairment and dementia while gathering information on important dementia risk factors. It achieves this by integrating the analyses of important risk factors such as depression and anxiety with cognitive screening tests in a conversation interface. It is important that the methods used within this app are accurate and easy to integrate within the wider structure of the app.

Both the 9-item Patient Health Questionnaire for depression (PHQ-9) and the 15-item Geriatric Depression Scale are widely used in clinical settings to screen for depression in older adults [8,9]. However, despite being shorter, PHQ-9 performs at least as well as the Geriatric Depression Scale in screening older adults across multiple populations, and therefore, we decided to incorporate PHQ-9 into the app [10,11]. The optimal cutoff point for the diagnosis of depression in PHQ-9 is ≥10 with an associated sensitivity and specificity of 88% [8]. Similarly, the 7-item Generalized Anxiety Disorder Scale (GAD-7) assessment has been used for anxiety screening with high sensitivity (92%) and specificity (76%) in those of working age with cutoff points ranging from ≥7 to ≥10 [12-14]. Further research has established effectiveness in screening older adults, with lower cutoffs of 5 recommended for better sensitivity [12,13].

Although these questionnaires are individually brief, they can become lengthy when nested within an app that aims to screen for multiple other risk factors and utilize multiple tests. Longer questionnaires cause higher rates of fatigue and dropouts, and hence, we aimed to limit the duration of total software use to 5 minutes [15]. This is especially key for individuals with affective disorders who are likely to experience deficits in attention, concentration, motivation, and fatigue [16,17]. We therefore considered PHQ-4 that combines PHQ-2 (which consists of the first 2 questions of PHQ-9) and GAD-2 (which consists of the first two questions of GAD-7) [18]. Although this shortens the time spent on the questionnaires, PHQ-4 does not have a severity scale, and commonly used cutoffs can result in prioritizing sensitivity or specificity at the expense of the other [19]. Consequently, this requires follow-up questions; for example, completing the whole PHQ-9 following a positive screen on PHQ-2 [20]. We wanted to develop a method that would have both the brevity of PHQ-4 and the accuracy of the longer PHQ-9/GAD-7 in addition to a severity scale. To achieve this, we adapted PHQ-4 by adding questions about factors normally covered in the longer questionnaires. We trained machine learning models that used the wide range of additional information already collected in Mindstep. Therefore, this study aims to assess the performance of our models when benchmarked against full-length standardized PHQ-9 and GAD-7 questionnaires. If performance was equivalent to that of these longer questionnaires, this would enable the app to have a screening method of equivalent efficacy while minimizing completion time.

## Methods

### App Data Collection and Users

Data of users of the Mindstep app were used for this study in a convenience sample. The app consists of a 5-minute conversational style questionnaire where information on common dementia risk factors is gathered. Cognitive performance is assessed through modified versions of 2 common cognitive tests: the Stroop test and Symbols Digit Modalities test. The dementia risk factors queried include medical history, age, alcohol consumption and dependency, concussion, smoking, and self-reported functional impairment. Analogous to medical consultation, further screening is then performed in response to answers that would elicit concern. Only those who reported feeling depressed or tired were screened with PHQ-9, while only those who reported feeling anxious or worried were screened with GAD-7. As a control group, for a short time, those who answered that they felt fine or great were also screened for anxiety. Apart from age, no other personal information was gathered from users.

### New Question Design 

**Figure 1:**
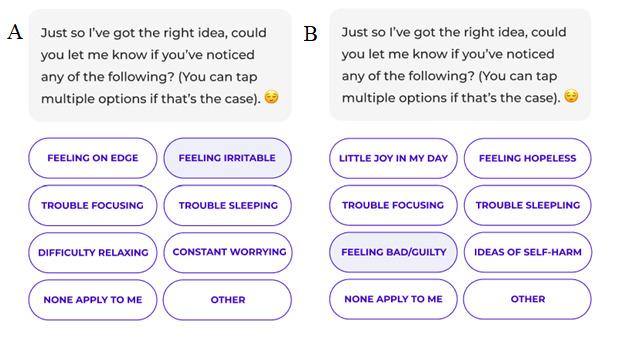
(A) Screenshot of the Mindset Anxiety Question. (B) Screenshot of the Mindset Depression Question.

New questions were created based on the longer versions of PHQ-9 and GAD-7, each for depression (Mindset Depression Question [MDQ]) and anxiety (Mindset Anxiety Question [MAQ]) to encompass symptoms of depression and anxiety in the Diagnostic and Statistical Manual of Mental Disorders that would normally be excluded from the shortened questionnaires. In 1 question, users were asked to select in a binary manner if any of several options applied to them—a method of collecting a wide range of information in a rapid manner. The questions are shown below (Figure 1). A mixture of categorical and continuous features from the app was used. The features were selected by unsupervised recursive feature elimination. Both models included age, alcohol dependency (as assessed by CAGE) [21], and a functional impairment question. For the PHQ models, the MDQ, PHQ-2, alcohol/drugs/smoking (currently/past/never), weekly alcohol consumption in drinks (<3/4-7/7-14/>14), feeling (depressed/tired), and joke data (yes/no/didn’t get it) were included. For the GAD models, MAQ, GAD-2, and the cognitive scores (MStroop/MSymbols) were included.

### Benchmarks

Outcomes of interest were compared to those of the full-length PHQ-9 and GAD-7. For the binary classification task, a cutoff value of ≥10 was used in both cases to represent commonly used screening cutoffs for these tools [14,22]. The total PHQ-9 and GAD-7 scores were also used for the regression task where the full-length screening score was predicted from PHQ-4 plus Mindset features.

### Data Preprocessing and Analysis

As part of the preprocessing pipeline, the categorical inputs were one-hot encoded. As only self-reported heavy drinkers were asked to complete the CAGE questionnaire, most users did not register a CAGE score and their score was assumed to be 0. For users who chose not to complete the Stroop or Symbols test, a mean value was used for their results. We divided the data into 80% training and a 20% holdout testing sets for PHQ (n=432,108) and GAD (n=408,103) with 10-fold cross validation. The holdout test set remained unseen throughout model training, hyperparameter tuning, and model selection.

### Machine Learning Models

Four distinct machine learning models were trained in both classification and regression task. The models used were logistic regression (linear regression was used in the regression task), support vector machines, TabNet [23], and extreme gradient boosted trees. The models were evaluated based on 10-fold cross validation scores (area under the curve [AUC] for classification, R^2^ for regression). The weaker models were discarded (AUC<0.9 or R^2^<0.7), and the final result was a median ensemble of the remaining models. Only the ensemble model was tested on the testing set. The performance on the training set of this ensemble model compared to that on the individual models can be found in Table S1 of Multimedia Appendix 1.

### Benchmarking

To guide interpretation and benchmarking of results, the final ensemble model was compared to logistic/linear regression models built using only the PHQ-2 and GAD-2 questionnaires. This was done on the unseen holdout test set. Confidence intervals and *P* values were generated to assess for the significance of differences by comparing model performance via a 1000-times bootstrap of the test set.

### Model Explainability

To interpret the predictions of the final ensemble model, model agnostic Shapley Additive Explanations (SHAP) scores were calculated to determine the relative feature importance [24]. SHAP scores determine which features are important to the model across the entire testing set and enable local interpretations such as why a particular prediction was made for a given user.

### Ethical Approval

This paper was a secondary data analysis of robustly anonymized data with minimal demographic information collected (only age) where there is no chance of data being linked to any individuals. On using the app, users agreed to transparent terms and conditions, which included having their data stored and anonymously used for further research. Therefore, ethical approval was not strictly required for this research. Out of an abundance of caution, we applied for and were granted retrospective ethical approval for the use of these data for research: West Midlands, Solihull Research Ethics Committee, Reference 21/WM/0202.

## Results

### Participant Data

Of the 2235 Mindstep users, 540 completed the PHQ and 511 completed the GAD. The mean age of the total Mindstep users was 50 (SD 14.1) years; for the PHQ subset, it was 49.3 (SD 13.1) years; and for the GAD users, it was 49.1 (SD 13.6) years. Of the 540 targeted users selected to take PHQ-9, 233 (43.1%) screened positive for depression. Of the 511 targeted users selected to take the GAD-7, 173 (33.9%) screened positive for GAD. These high rates likely represent the enriched selection of users who had already reported feeling negatively valenced emotions. Only a small number of users did not complete the Stroop (28/2235, 1.3%) or Symbols tests (26/2235, 1.2%) and had data imputed.

### Questionnaire Characteristics

**Figure 2:**
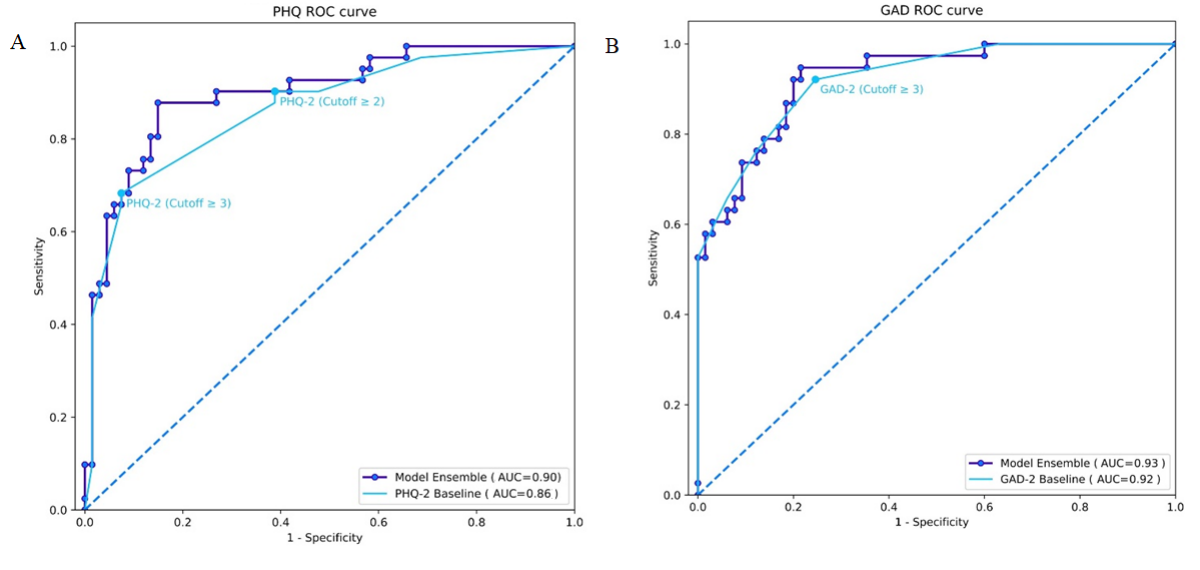
Receiver operating characteristic curve for prediction of (A) Patient Health Questionnaire for depression and (B) Generalized Anxiety Disorder Scale of the model ensemble and their respective baselines. GAD: Generalized Anxiety Disorder; PHQ: Patient Health Questionnaire; ROC: receiver operating characteristic.

All questionnaires had excellent reliability as measured by the Cronbach interitem correlation [25]: PHQ-9 (*α*=.84), PHQ-2 (*α*=.77), GAD-7 (*α*=.90), and GAD-2 (*α*=.84). The test set AUC for the ensemble model for PHQ-9 (0.90) was a significant improvement on the PHQ-2 baseline (difference 0.04, 95% CI 0.00-0.08; *P*=.02). The test set AUC for the ensemble model for GAD-7 (0.93) was equivalent to the GAD-2 baseline (difference 0.00, 95% CI –0.02 to 0.03, *P*=.42) (Figure 2). By altering thresholds, the sensitivity and specificity of the ensemble models can be optimized for particular situations. The selected optimal sensitivity and specificity for the PHQ model was 88% and 85%, respectively, achieving a good compromise compared to the highly sensitive PHQ-2 cutoff ≥2 (90% and 61%, respectively) or highly specific PHQ-2 cutoff ≥3 (68% and 93%, respectively). The positive and negative predictive values for the PHQ model were 78% and 92%, respectively. The sensitivity and specificity of the GAD model (95% and 78%, respectively) were substantively similar to the clinically used GAD-2 cutoff ≥3 (92% and 75%, respectively). The positive and negative predictive values for the GAD model were 69% and 96%, respectively.

### Regression Analysis

**Figure 3:**
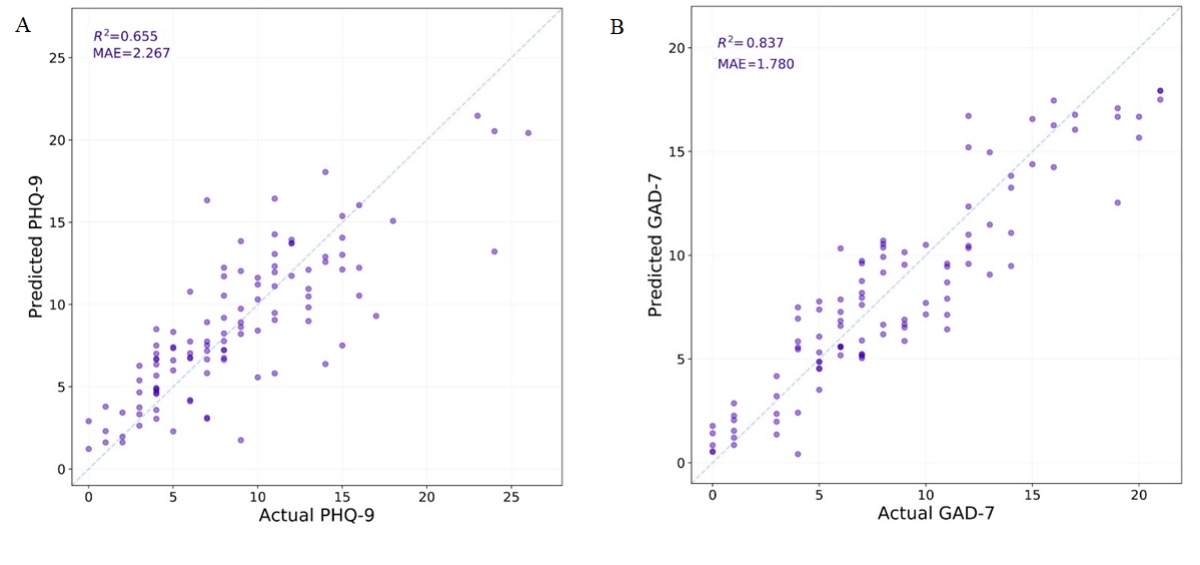
The regression model ensemble predictions for the test set (A) 9-item Patient Health Questionnaire for depression and (B) 7-item Generalized Anxiety Disorder scale scores. GAD-7: 7-item Generalized Anxiety Disorder scale; MAE: mean absolute error; PHQ-9: 9-item Patient Health Questionnaire for depression.

Figure 3 shows the regression model ensemble predictions for the test set PHQ-9 and GAD-7 scores. Both models (Figure 3) were able to achieve good prediction of the full-length questionnaire scores: PHQ-9 (R^2^=0.655, mean absolute error [MAE]=2.267) and GAD-7 (R^2^=0.837, MAE=1.780). The PHQ model showed a significant improvement in MAE over the PHQ-2 baseline (difference 0.35, 95% CI 0.06-0.65, *P*=.01) and a nonsignificant improvement in R^2^ (0.08, 95% CI –0.02 to 0.21, *P*=.06). The GAD model showed a nonsignificant improvement in MAE over the GAD-2 baseline (0.08, 95% CI –0.10 to 0.26, *P*=.20) and a significant improvement in R^2^ (0.04, 95% CI 0.01-0.08, *P*=.01) (Table S1 in Multimedia Appendix 1). For PHQ, by breaking the scores into categories of increasing severity [8], 0-4, 5-9, 10-14, 15-19, and ≥20, an intraclass correlation was calculated to be 0.76 (95% CI 0.67-0.83, *P*<.001). For GAD, categories of 0-4, 5-9, 10-14, and ≥15 were used [14]. An intraclass correlation was calculated as 0.87 (95% CI 0.81-0.91, *P*<.001).

### Feature Importance

**Figure 4:**
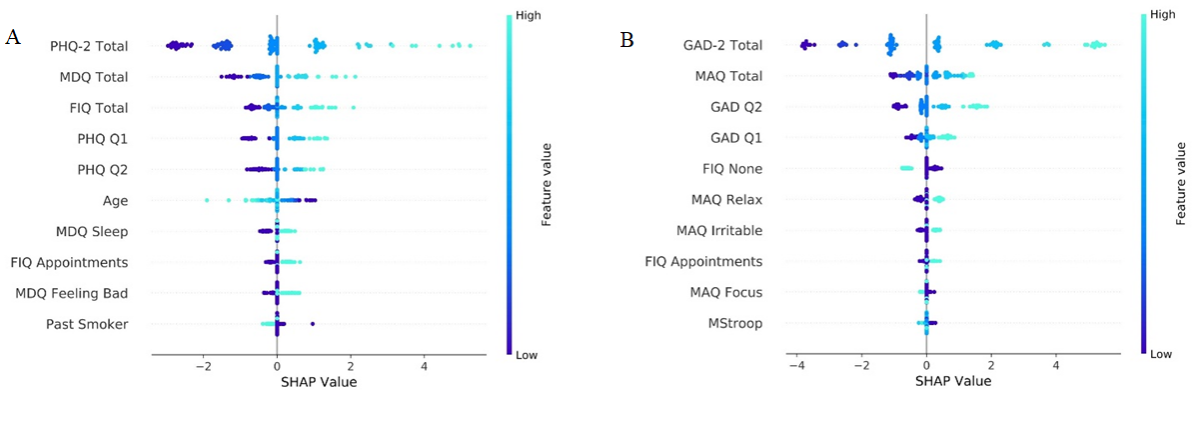
Beeswarm Shapley Additive Explanations summary plots of the 10 most important features as determined by Shapley Additive Explanations values for predicting the (A) 9-item Patient Health Questionnaire for depression and (B) 7-item Generalized Anxiety Disorder scale. Ordered by importance (top to bottom). The greater the magnitude of the Shapley Additive Explanations values, the larger the influence on the model with positive numbers, indicating the user is more likely to have the condition. FIQ: functional impairment question; GAD-7: 7-item Generalized Anxiety Disorder scale; MAQ: Mindset Anxiety Question; MDQ: Mindset Depression Question; PHQ-9: 9-item Patient Health Questionnaire for depression; SHAP: Shapley Additive Explanations.

Figure 4 shows the beeswarm SHAP summary plots of the 10 most important features as determined by SHAP values for predicting the PHQ-9 and GAD-7. The greater the magnitude of the SHAP values, the larger was the influence on the model with positive numbers, indicating that the user is more likely to have the condition. In both cases, the most important features in the prediction of the full-length questionnaires were the PHQ-2 and GAD-2 followed by the MDQ and MAQ, respectively. The functional impairment question was also shown to be important in both sets of models. Age and smoking were important in the depression models, and the Stroop test was important in the anxiety models.

## Discussion

### Principal Results

We have shown that the combination of PHQ-4, additional questions, and risk factor information are able to accurately predict the severity score of the longer questions with an R^2^ of 0.655 on PHQ-9 and R^2^ of 0.837 for GAD-7. This is a novel finding, as previous studies have only looked at agreement between the binary cutoffs of the shorter questionnaires compared to that of the longer. This suggests that even these ultrashort questionnaires may be responsive to change, although this will need to be explored in future work. In addition to this, compared to using the PHQ-2 alone, our model achieves significantly better performance on both classification and regression models. The SHAP analysis suggests that the MDQ and MAQ can capture some of the variance missed by the shorter PHQ-4. The benefit of our model is less clear in anxiety with little difference compared to utilizing the GAD-2 alone. This may be in part, as GAD-2 alone achieves very high performance on both the binary classification and regression task. This is in line with meta-analyses, which show that GAD-2 achieves very similar performance to GAD-7 [12-14]. The strength of our developed model for depression is not just an enhanced accuracy of prediction but also our ability to choose any threshold to best balance sensitivity and specificity. This enables us to choose a cutoff that best balances sensitivity and specificity rather than having to choose between a PHQ-2 cutoff, which prioritizes a high sensitivity or specificity. Furthermore, the fact that we collect age will enable us to personalize the cutoff for screening in line with evidence by using less stringent cutoffs in older adults to maximize sensitivity [12,13]. Our regression model with good intraclass correlations enables us to sort users into multiple categories. For example, initial validation studies of PHQ-9 demonstrated that while 10 represented the best cutoff for sensitivity and specificity, higher scores had much better discriminative values with scores above 15 highly specific for depressive disorders and 10-15 representing an important grey zone [8]. Therefore, sorting users in multiple categories such as unlikely (<10)/possible (10-15)/probable (>15) depression is achievable and will assist in optimizing the accuracy of advice we can offer. Data suggest that longer questionnaires incur more fatigue and dropout [15]. This is especially important, as our app is self-administered and there is no clinician to encourage the user. The benefit of using ultrashort questionnaires is that it allows for many different risk factors to all be assessed with a single app and in a single sitting without significant fatigue and dropout. This enables a comprehensive review of many risk factors for dementia.

### Limitations

Although these questionnaires are filled out unsupervised on an app, the validity of the computerized forms of PHQ-9 and GAD-7 has been demonstrated to be valid across format types [26], and the excellent reliability achieved in this study negate this as an issue. A limitation of this study is the use of PHQ-9/GAD-7 as our ground truth. This is an indirect measure compared to clinician-assessed diagnosis or structured diagnostic questionnaires such as the Structured Clinical Interview for Diagnostic and Statistical Manual of Mental Disorders-IV (SCID). This both means that we cannot train our model on a definite diagnosis of depression. However, previous studies that have looked for proxies of depression diagnosis achieved AUCs of 0.77 [26] and 0.79 [27], which are low compared to the accuracy of the full PHQ-9 (AUC 0.87) [28]. This suggests that PHQ-9 is a valid ground truth to use. An additional limitation of the use of PHQ-9/GAD-7 as our ground truth means that our model can never outperform these questionnaires. Considering that we collect data on a wider variety of factors including functional impairment, it may be that comparison to diagnostic measures would allow enhanced performance. Indeed, by capturing functional measures, including cognitive performance and self-perceived deficits, our app captures important elements missing from the PHQ-9 and GAD-7 questionnaires. Interestingly, the SHAP analysis shows that the questions around functional deficits are especially important in the PHQ-9 model, suggesting this information is important to the model’s outperformance of the PHQ-2 alone. The next step to address this limitation would be to use the app in a population with gold standard validated measures of mental illness such as a SCID conducted by a mental health professional. This would both allow refinement of factors in the model and allow assessment of true sensitivity and specificity when it comes to the diagnosis of depression. However, it is important to note that the intent of the app is to use this function strictly in a screening and not a diagnostic role, with identified individuals being signposted for further assessment by their primary care doctors. Therefore, correlation to an already extremely well-validated questionnaire is likely to be adequate for its purpose.

Owing to the need for the app to collect anonymized data, we do not have basic demographic data such as gender, ethnicity, sociodemographic status, or educational background, which is an important limitation of this study. This makes it difficult to explore biases in the model, which may result in differing performance across demographic groups. Further work will need to be done to explore the algorithm’s performance in a diverse range of groups to guard against differential performance. In addition, this limitation makes it hard for us to generalize these findings to a specific group outside of users of our app. However, the MAQ/MDQ are based on widely accepted symptoms that are likely to maintain their validity outside of this setting. Another limitation of this study was the relatively small sample size; future investigations could expand to larger data sets. In this vein, an important future step is to test the effectiveness of this algorithm in a setting with participants with well-labelled characteristics—an essential follow-on to the initial validation [29]. A future trial is planned to assess this in a group of older adults, an especially important group, since evaluating depression and anxiety are in the context of dementia risk factors and screening.

### Conclusion

In summary, our results suggest that by using the PHQ-4, in line with the other measures collected in the Mindstep app, we can achieve accuracies similar to full-length PHQ-9 and GAD-7 questionnaires. This suggests that the app can be used to reliably screen for these conditions. Further work in populations with validated diagnoses whose demographics are known will further strengthen the evidence underlying these models.

## Appendix

Multimedia Appendix 1 Supplementary information on the performance of the machine learning models in the training set.

## Abbreviations

AUC: area under the curve
CAGE: Cut, Annoyed, Guilty, and Eye Questionnaire
GAD-7: 7-item Generalized Anxiety Disorder Scale
MAE: mean absolute error
MDQ: Mindset Depression Question
MAQ: Mindset Anxiety Question
PHQ-9: 9-item Patient Health Questionnaire for depression
SCID: Structured Clinical Interview for Diagnostic and Statistical Manual of Mental Disorders
SHAP: Shapley Additive Explanations
