# Supplementary material for: Optimizing Existing Mental Health Screening Methods in a Dementia Screening and Risk Factor App: Observational Machine Learning Study
Source: JMIR Form Res. 2022 Mar 22;6(3):e31209. doi: 10.2196/31209 (PMC8984825; doi:10.2196/31209)
Supplement: Multimedia Appendix 1 [file formative_v6i3e31209_app1.docx]

Supplementary Materials

**Table S1**

|  | Logistic/Linear Regression | Extreme Gradient  Boosted Trees | TabNet | Support Vector Machine | Ensemble |
| --- | --- | --- | --- | --- | --- |
| Patient Health Questionnaire for depression-9 items - Binary predictor | 0.88 | 0.92 | 0.93 | 0.86 | 0.95 |
| Patient Health Questionnaire for depression-9 items - Regression | 0.76 | 0.69 | 0.72 | 0.72 | 0.77 |
| Generalized Anxiety Disorder scale-7 items - Binary predictor | 0.89 | 0.93 | 0.93 | 0.88 | 0.97 |
| Generalized Anxiety Disorder scale-7 items - Regression | 0.84 | 0.81 | 0.79 | 0.81 | 0.86 |

Performance of individual models and final ensemble model on the training sets as measured by Area Under the Curve
